# Problem solving is embedded in context… so how do we measure it?

**DOI:** 10.3389/fpsyg.2024.1380178

**Published:** 2024-05-17

**Authors:** Katherine T. Rhodes, Lindsey E. Richland, Lucia Alcalá

**Affiliations:** ^1^Language Variation and Academic Success (LVAS) Lab, School of Education, University of California, Irvine, Irvine, CA, United States; ^2^Science of Learning (SoL) Lab, School of Education, University of California, Irvine, Irvine, CA, United States; ^3^Culture and Social Action Lab (CaSA), Department of Psychology, California State University, Fullerton, Fullerton, CA, United States

**Keywords:** problem solving, measurement, information relevance, ecological validity, cultural relevance

## Abstract

Problem solving encompasses the broad domain of human, goal-directed behaviors. Though we may attempt to measure problem solving using tightly controlled and decontextualized tasks, it is inextricably embedded in both reasoners’ experiences and their contexts. Without situating problem solvers, problem contexts, and our own experiential partialities as researchers, we risk intertwining the research of information relevance with our own confirmatory biases about people, environments, and ourselves. We review each of these ecological facets of information relevance in problem solving, and we suggest a framework to guide its measurement. We ground this framework with concrete examples of ecologically valid, culturally relevant measurement of problem solving.

## 1 Introduction

As of writing this perspective piece, there exist pockets of the world with ubiquitous internet, fingertip access to generative artificial intelligence, and engagement with global news and commerce, while other humans grapple more regularly with local subsistence farming, climate change, and family social relationships. These are abstracted points of comparison among the incredibly varied social and cultural contexts in which human reasoners must draw from information in their environments to notice problems that need resolution, find relevant information from which to make inferences, and execute problem solutions. This wide variance highlights the deep theoretical and practical challenges of characterizing and measuring problem solving as a pragmatically-grounded, cognitive construct.

In this perspective piece, we focus on measurement theory for gathering data on the complex cognition that governs humans’ everyday lives, focusing on problem solving in specific. Problem solving broadly encompasses human goal-directed behaviors (Newell and Simon, 1972). Though problem solving may include a variety of goal structures in everyday living (from solving a mathematical problem in a formal educational setting to identifying the need for housework in one’s family context), it is often measured with highly abstracted tasks that attempt to decontextualize problems from the specific in favor of the universal (Jukes et al., 2024).

We posit that measurement of problem solving with recognition of the deeply intertwined nature of reasoning with one’s context necessitates that we must center (a) the experiences and perceptions of the problem solver, (b) the context in which problem solving is being observed, and (c) the lens through which we as observers are interpreting problem solving. Each of these ecological facets influences our interpretations of problem-solving behavior, and each is socioculturally bound. Without situating problem solvers, problem contexts, and our own experiential partialities as researchers, we risk intertwining the research of information relevance with our own confirmatory biases about people, environments, and ourselves. We review each of these ecological facets of information relevance in problem solving, and we suggest a framework to guide ecologically valid, culturally relevant measurement.

## 2 Centering the relevant experiences and perceptions of the problem solver

We naturally use our problem-solving resources to attend to experientially relevant information, and thus, problem-solving tasks are socioculturally bound to problem solvers (Oyserman, 2011, 2016). Our measures of problem solving broadly reflect different attentional patterns that are based on prior developmental experiences which differ depending on socialization (Newell and Simon, 1972; Ericsson et al., 1993). Broadly speaking, this means that the measurement of problem-solving tasks is closely tied to the particular experiences of problem solvers.

Consider, for example, the famous marshmallow experiment (Mischel, 1961; Mischel and Metzner, 1962; Mischel and Ebbesen, 1970). In this lab-based experimental task, children are given a marshmallow and told that they may eat the treat immediately or wait an unspecified amount of time and receive additional marshmallows as a reward. Performance on the marshmallow task has typically been interpreted to indicate ability to delay gratification, (i.e., inhibitory control), and it has been linked to later academic performance, self-confidence, likelihood of subsequent substance abuse, and a variety of other outcomes (Mischel et al., 1988, 1989; Shoda et al., 1990; Ayduk et al., 2000). Thus, the researcher-identified problem of the marshmallow task is (1) the identification of the marshmallow as a reward, (2) the decision to engage in a desired goal-oriented behavior (waiting) to obtain the reward, and then (3) the execution of the desired goal-oriented behavior (engaging inhibitory control in order to wait).

However, some researchers have raised concerns about the interpretation of performance on the marshmallow task, in particular, questioning what we might reasonably infer about the relevant pieces of information that children use to execute decision-making about whether or not to wait. For example, Kidd et al. (2013) found evidence that children’s rational decision-making about the reliability of the experimental environment (and by implication, their prior experiences with reliable and unreliable environments) may also influence their decisions to delay gratification comparably to their individual differences in capacity for self-control.

Other researchers have noted that the “The Marshmallow Test” may simply be a culturally loaded problem-solving task with narrow expectations about children’s behavior and ways of solving the problem. For example, Yucatec Maya children are often engaged in real-life productive activities, are motivated to contribute, and allowed to take the initiative to solve problems they encounter (Gaskins, 2020; Cervera-Montejano, 2022). When encountering novel problems, they are expected to be attentive and learn by observing others and not just by listening to verbal instructions (Alcalá et al., 2021). However, when Gaskins tried to replicate this study with Yucatec Maya children, she found that none of the six children she tested earned the second marshmallow (Gaskins and Alcalá, 2023). Two of them ate the treat, and four of them left the room. Gaskins attributes their marshmallow task performance differences to the cultural assumptions in the methodology, such as the expectation that children will obediently attend to and follow adult’s instructions. The children who left the room did not leave because they were tempted to eat the marshmallow – which assumes poor self-regulation – but they left because “they saw no ‘good reason’ to sit alone in a room for a long time doing nothing, rejecting the basic premise of the task.” (p. 8). Gaskins and Alcalá (2023) results illustrate that participants’ perceptions about adult authority, expectations for child compliance, and familiarity with verbal instructions are also relevant and often overlooked aspects of the marshmallow experiment.

The marshmallow task illustrates that the same contextual cues may be interpreted very differently by different experimental participants because prior experiences influence our expectations, beliefs, and ultimately, our mental representations of the problems we are solving. Lab-based problem-solving tasks like the marshmallow task have the advantages of being tightly controlled, but they are also decontextualized, adult-generated, and assume child compliance based on the lived experiences and rules familiar to White, middle-class children (Jukes et al., 2024). Examining psychological constructs and tasks across contexts can help illuminate characteristics of problem-solving tasks that may be reflecting culturally-derived experiences and socialized expectations.

## 3 Centering the sociocultural context in which problem solving is being observed

The sociocultural context in which problem solving is being observed helps define the parameters of the problem being solved, which in turn influences the pieces of information that may be relevant to its effective solution. Consider, for example, the sociocultural norms that contextualize children’s helping behaviors in their homes and communities. Helping behaviors are also goal-oriented, problem-solving behaviors that are prosocial in nature – they require the identification of a social problem (the need for help to occur), the formulation of a solution (selecting the kind of help that will remedy the identified issue), and the execution of a solution (engaging in helping until a desired goal has been reached). In many Western, educated, industrialized, rich, and democratic (WEIRD) societies, children are viewed as the recipients of help rather than as independent, helpful agents in their communities (Ochs and Izquierdo, 2009). However, in communities where children are socialized to provide substantial contributions to their families, taking the initiative to help with complex household tasks and to assist during community celebrations, the contextual expectations around problem solving might be quite different (Rogoff, 1990; Chavajay and Rogoff, 2002).

During a visit to Yucatan, Alcalá (2023) observed how children are given extensive amounts of autonomy to decide how to spend their time, including helping with household work and engaging in unstructured play activities. In this context, children are expected to notice when there is a problem and act accordingly to find the appropriate solution (Alcalá and Cervera, 2022). Mothers state that children need to learn to be autonomous because they might not always be with adults or others that can help them, and need to learn to solve the problems they encounter.

Alcalá et al. (2021) asked children why they help at home and the majority of them reported that they help because helping is a shared responsibility of all family members, they help because they like to help, or they help because they notice work that needs to be done. The cultural expectations to be attentive to their surroundings, to be autonomous and self-directed in choosing activities, and to notice work and problems in need of solution is key in how children in this community learn to solve problems. For example, children notice that there are some dirty dishes and will go and wash the dishes, or they might notice that the plants need to be watered.

The shared responsibility to help and solve problems, opens other opportunities for children to identify and solve problems in their communities. For example, children might notice or hear about a family member who is ill, and they volunteer to help with chores that would normally be done by the ailing adult as illustrated by “*Soledad*” (Chan Cah, age 10) *“my mom’s foot hurt and that is why I help”* (Alcalá et al., 2021, p.).

Furthermore, when asked what would happen if they do not help, about half of the participants responded in a way that reflected a community-minded way of solving problems. Children indicated that if they do not help, for example with washing the dishes, then the pile of dirty dishes will get bigger and then someone else would have to wash the dishes. Likewise, if a child does not help with the *milpa* (corn field) there might not be enough corn for the family.

In this context, where children are allowed to be present and observe almost all of the activities of the household and community, children are expected to become interested and notice when someone needs help (López Fraire et al., 2024). Children are trusted enough to solve certain problems on their own, or know when to find help, as they are becoming competent members of their communities.

The sociocultural context helps to dictate what is a problem, who is affected by the consequences of the problem, and who is allowed, expected, and empowered to solve the problem. Importantly, the sociocultural context also determines the level at which problems exist - Not all problems belong to the individual as is often assumed in highly individualistic societies (Oyserman et al., 2002; Arieli and Sagiv, 2018). In many problem-solving contexts across the world, problems, their consequences, and the responsibility for solving them belong to groups and communities of problem-solvers (Lasker and Weiss, 2003).

## 4 Discussion

### 4.1 How do we measure problem solving?: considering the lens of the research observer

For many researchers, the measurement of problem solving may appear to be a primarily methodological issue at first glance (Messick, 1981). We create tasks, observe individual differences in task performance, and assign interpretations for those differences. The measures are assumed to be objective, empirical, quantitative metrics of performance – Child X ate marshmallow Y after Z minutes of waiting, therefore failing to delay gratification with additional marshmallows (see Mischel et al., 1988). However, without the guidance of strong theoretical postulates about constructs, and without clear links between theoretical postulates and the measures designed to capture constructs of interest, we are asking our measures to do the work of specifying larger theoretical models (Borsboom, 2005).

Our measures reflect our theoretical dispositions, and our theories reflect ourselves. The lens through which we generally interpret cognitive development is culturally misaligned with the majority of the world’s problem solvers and problem-solving contexts, and our measures of problem solving reflect that epistemological misalignment. As researchers who are primarily from Western, educated, industrialized, rich, and democratic societies, our lens for understanding and measuring human behavior is WEIRD (Henrich et al., 2010). Problem solving is no exception and has traditionally been measured in WEIRD ways with WEIRD problem solvers, which can misrepresent developmental phenomena that may not replicate with children from other sociocultural backgrounds or lived contexts (e.g., for evidence of this in the above marshmallow task, see Watts et al., 2018). These traditional measures of problem solving do not account for potential sociocultural differences in information processing that can derive from the nature of the task requirements to the cultural context of how children should speak with adults. For problem solving measures, one must step back to consider that even the definition for what constitutes a problem that a participant has the authority to solve is cultural, with measures tending to be based on WEIRD researchers’ known context, which can lead to bias then in solution rates and participants’ engagements. Thus, it is unsurprising that children who are not from WEIRD communities or who are marginalized within WEIRD societies may perform differently on traditional measures of problem solving (see for example, Miller-Cotto et al., 2022).

If our aim is to capture problem solving in ways that have meaningful implications for the real world information processing, we need to measure problem solving in ways that are culturally relevant for broad populations of children. This aim is critical for problem-solving research, and it necessitates an epistemological (and possibly an ontological) recentering of our measurement of problem solving.

### 4.2 Framework for ecologically valid, culturally relevant measurement

There is a growing push to measure human problem solving “in context,” in ways that are ecologically valid (see for example Burgess et al., 2006; Miller and Scholnick, 2015); however, contextualized tasks can still evidence the same biases that create validity issues for traditional, abstract, decontextualized tasks. The field has a pressing need for a framework that helps researchers to evaluate problem-solving tasks in ways that consider their relevant features from the perspective of diverse learners. To support an evolution in the fields of reasoning and problem solving that better centers tasks and measurement on the abilities executed by reasoners in their everyday worlds, we propose a set of questions that researchers can ask when developing a task to better ensure relevance and alignment between test participants, researchers, and the interpretation of empirical data.

#### 4.2.1 Understanding problem solvers’ relevant experiences

##### 4.2.1.1 How are reasoners perceiving the problem?

✓ Assume that the problem solver’s solution is predicated on the kind of mental representation she has formed about the problem.
🛇 Avoid assuming that problem solvers perceive the same goal-structure, have the same mental representation of the problem, or have the same reasoning and approach to solving the problem. Problem solvers are NOT necessarily attending to the researchers’ desired matrix of information when thinking about the problem.
★ Forexample, Rhodes et al. (under review) research on the mathematical problem solving of African American children who use African American English dialect (AAE; a cultural dialect of American English) explored the types of errors that children make on various arithmetic problems as a function of both item formatting and the density of children’s AAE dialect usage. The very exploration of this research question runs counter to the assumption that word formatting and children’s home language would have no impact on African American children’s mental representations of problems and strategic approaches to solving them. Results suggested that children’s strategic errors occurred as a complex interaction between word problem formatting and children’s AAE dialect density, effectively challenging the assumption that word problems would elicit language neutral mental representations with African American children whose home and community language systems were linguistically distanced from them.

##### 4.2.1.2 What does unexpected or “non-normative” task performance mean?

✓ Assume that divergence from a normative expectation is not necessarily indicative of pathology or lack of skill.
🛇 Avoid assuming that we manage attentional resources during problem solving in one, normative way. In particular, avoid the assumption that problem solving is maladaptive – instead, look for the adaptive response in the way that you interpret the problem solving.
★ Forexample, a child who does not concentrate fully on a problem solving task they have been given, but instead is also directing attention toward monitoring the experimenter’s actions and conversations with another child, may be exhibiting highly culturally appropriate and intentional resource allocation to ensure they are not missing a need to learn new relevant information or assist the experimenter (e.g., Correa-Chávez et al., 2005). Challenging the assumption that the management of attentional resources should happen in one, normative, culturally-sanctioned way, creates the opportunity for researchers to recognize important sources of cultural variance in otherwise invisible aspects of task construction (i.e., prosocial attentional engagement as a means of identifying information relevance).

#### 4.2.2 Considering socio-cultural contexts of problem solving

##### 4.2.2.1 Where do problems occur?

✓ Assume that there are no neutral contexts for problem solving. The “lab” (a tightly controlled experimental context) is not, in fact, neutral.
🛇 Avoid assuming that the most meaningful problems we solve occur in formal educational settings or in tightly controlled experimental settings.
★ Forexample, in his landmark study of Brazilian child candy sellers, Saxe (1988) used a multimethod paradigm to observe and query the naturalistic mathematical behaviors of children in- and out-of-classroom mathematics problem-solving contexts. In challenging the assumption that normative mathematical problem solving only develops in formal educational contexts, he observed that the skills children used in their street vending activities did not necessarily transfer to their school contexts and vice versa, and importantly, that children who were quite adept at using mathematics in their real-world vending activities were not necessarily able to translate their skills toward high-achievement on formal educational tasks (Saxe, 1988).

##### 4.2.2.2 For whom is the problem consequential? and relatedly, who is empowered to solve the problem in this context?

✓ Assume that problem solving is not necessarily an individual sport - individuals, groups, and communities may identify problems, problem consequences, and problem solvers very differently.
🛇 Avoid assuming that problem solving should only be conceptualized and measured at the individual level. Similarly, avoid the assumption that cultural expectations for problem solving converge around efficiency (i.e., quickly and accurately; careless mistakes may have important consequences beyond an individual).
★ Forexample, when asked why they help with household chores, most Yucatec Maya children mentioned that if they did not do the chore, this would create more work for their parents or cause harm to others including younger siblings or aging adults (Alcalá and Cervera, 2022). In challenging individualistic assumptions about measuring problem solving, these researchers were able to capture children’s mental representations of problems and problem consequences as belonging to the entire household, rather than assigning the responsibility for problem solving to a household’s individual members.

#### 4.2.3 Evaluating researchers’ perspectives of problem solving

##### 4.2.3.1 How does the observer’s positionality influence the evaluation of problem solving?

✓ Assume that positionality is something we can and should acknowledge, particularly if we are evaluating the problem-solving abilities of others.
🛇 Avoid assuming that researchers have the same positionality as research participants or groups to whom research is generalized (see for example, Bilgen et al., 2021; Patton and Winter, 2023).
★ Forexample, Patton and Winter (2023) provide a detailed and reflexive account of researcher positionality and decision-making in engaging in an observational study with preschool-aged children. These researchers consider the use of a teddy bear named “Ted” as an elicitation tool for gathering information about children’s perspectives and contextual experiences of early childhood educational settings. In examining their own positionalities, the authors were able to interrogate the inherent power structure between adults and children in traditional research participation paradigms. This consideration of positionality helped inform the researchers’ decision to embed “Ted” into children’s preschool contexts in meaningful ways that allowed children to engage with him as a peer, including him in activities and songs, helping him, or even explaining mistakes to him in the role of experts.

##### 4.2.3.2 What can we infer from a reasoner’s problem-solving actions?

✓ Assume that the interpretation of problem-solving actions will be influenced by the problem solver, the context for problem solving, and the research observer.
🛇 Avoid assuming that a particular measurement instrument is contextually neutral or culturally unbiased. It is critical that we acknowledge the fact that measurement instruments are also NOT free of positionality. They exist in the context of larger epistemologies that influence their design, application, and interpretation.
★ Forexample, many laboratory tasks assume that children are familiar with and willing to follow adults’ instructions, even if the tasks do not accomplish readily apparent goals such as care or feeding. These tasks then may yield biased conclusions when used with children from communities which value autonomy over decision-making, specifically where respect for children’s ability to decide about their participation in activities means they are not required to obey adults; such children may perform poorly on these types of tasks or refuse to follow the researcher’s instructions (Jukes et al., 2024).

## 5 Conclusion

We argue that problem solving is fundamentally and inextricably tied to deeper, often implicit, questions of epistemology, which need to be made explicit to facilitate its meaningful measurement. This philosophical work cannot be undertaken during methodological decision-making alone. Rather, if we hope to validly and reliably measure problem solving, we must also formulate strong theoretical positions about what it is, how it operates across various contexts of interest, and how we may observe it – all of which must be integrated and mapped onto specifications of our models of measurement. For as illustrated by the difficulties in interpreting performance on the marshmallow task, children with various prior experiences, in various sociocultural contexts, may have vastly different experiences of problem-solving the same task.

To be clear, rigorous measurement of information relevance in problem solving does not require that we abandon the empirical tenets of modern measurement theory. Nor does it require the rejection of the thoughtful positionality critiques of critical theorists. Rigorous research of problem solving requires the careful consideration of these seemingly irreconcilable epistemologies and, where possible, the integration of them in research design and interpretation.

Measuring problem solving “in context” does not necessarily remedy the issue of culturally biased measurement because contextualized for one group may be decontextualized (and biased) for another group. The wide variance in our experiences and contexts may necessitate admission that there may not be a perfect, unbiased measure of human problem solving, and the best measure for one’s particular research perspective will likely have shortcomings. Still, rigorous measurement of information relevance in problem solving demands that we acknowledge these shortcomings and interpret performance with sensitivity to them. The authors recognize that this process is not easy. We grapple with this in our own work; however, we believe that the process of grappling with these epistemological issues is central to the evolution of our research.

## Data availability statement

The original contributions presented in the study are included in the article/supplementary material, further inquiries can be directed to the corresponding author.

## Author contributions

KR: Conceptualization, Funding acquisition, Methodology, Project administration, Resources, Validation, Visualization, Writing – original draft, Writing – review & editing. LR: Conceptualization, Funding acquisition, Methodology, Project administration, Resources, Validation, Visualization, Writing – original draft, Writing – review & editing. LA: Conceptualization, Funding acquisition, Resources, Validation, Visualization, Writing – original draft, Writing – review & editing.
